# Water extract of *Semecarpus parvifolia* Thw. leaves inhibits cell proliferation and induces apoptosis on HEp-2 cells

**DOI:** 10.1186/s12906-018-2142-8

**Published:** 2018-03-05

**Authors:** Preethi Soysa, Panchima Jayarthne, Imali Ranathunga

**Affiliations:** Department of Biochemistry and Molecular Biology, Faculty of Medicine, Colombo, Sri Lanka

**Keywords:** *Semecarpus parvifolia*, Traditional medicine, Antiproliferative activity, Apoptosis

## Abstract

**Background:**

*Semecarpus parvifolia* Thw is used as an ingredient of poly herbal decoctions to treat cancer in traditional medicine. The present study aims to investigate the antiproliferative activity on HEp 2 cells by the water extract of *S. parvifolia* leaves and to evaluate potential mechanisms.

**Methods:**

The plant extract was exposed to *S. parvifolia* for 24 hours and antiproliferative activity was quantified by Sulforhodamine B (SRB), 3-(4, 5-dimethythiazol-2-yl)-2, 5-diphenyl tetrazolium bromide (MTT) and Lactate dehydrogenase (LDH) assays. Morphological changes were observed after staining cells with ethidium bromide/acridine orange (EB/AO) and Giemsa dye. Comet assay was performed to evaluate the DNA damage. The toxicity of the plant extract was determined by brine shrimp lethality assay.

**Results:**

*S. parvifolia* leaves reduced the cell proliferation in a dose and time dependent manner. A two fold increase in NO level was observed at higher concentrations. Morphological changes characteristic to apoptosis were observed in light microscopy, Giemsa and EB/AO stained cells. Fragmented DNA further confirmed its capacity to induce apoptosis. No lethality was observed with brine shrimps.

**Conclusion:**

The results suggest that *Semecarpus parvifolia* Thw induces apoptosis in HEp-2 cells through a NO dependent pathway.

## Background

Cancer is one of the leading cause of death worldwide [1]. Mutations in DNA or modification of epigenetic processes transform normal cell into a malignant cell resulting an uncontrolled cell division. [2]. Over the years, many natural product-based drugs have been introduced. Most of the marketed drugs for cancer therapy are unmodified natural products or their semi-synthetic derivatives or synthesized molecules based on natural product compound pharmacophores [3]. Vinblastine, vincristine, vinorelbine (vinca alkaloids), etoposide, teniposide, etoposide phosphate (epipodophyllotoxin lignans), paclitaxel, docetaxel (taxane diterpenoids), and toptotecan, irinotecan (camptothecin, quinoline alkaloid derivatives) are such plant derived secondary metabolites used in the treatment of cancer [3–5].

Most of the drugs used in traditional medicine are poly herbal mixtures. *Semecarpus parvifolia* is a component of some of the poly herbal drugs. The gum of its bark, seeds and leaves are used in the treatment of cancer in traditional medicine. *S. parvifolia* is an endemic plant to Sri Lanka which belongs to the Family of Anacardiaceae. Most of the studies on medicinal effects and toxicity have been evaluated for *Semecarpus anacardium* Linn [6–8]. *Semecarpus anacardium, Semecarpus obovate* and *Semecarpus subpeltata* are used as substituents for *Semecarpus parvifolia* [9]. Previous studies have shown that *Semecarpus anacardium* possesses antiproliferative activity against breast cancer cell lines [10]. Anticancer potency in hepatocellular carcinoma has been demonstrated with milk extract of nuts of *Semecarpus anacardium* Linn. in rats [11]. It has been found that, water extract of *S. parvifolia* leaves has a high capacity to scavenge free radicals in vitro [12]. Studies on anticancer activity of *S. parvifolia* is lacking and this study was designed to evaluate the antiproliferative activity and the mode of cell death of *S. parvifolia* Thw.

## Methods

### Materials and Equipment

The chemicals and cell culture reagents were purchased from Sigma Chemicals Co. (P.O. Box 14508, St. Louis, MO 63178 USA) or Fluka (Flukachemie GmbH, CH-9471 Buchs) unless otherwise stated. Lactate Dehydrogenase (LDH) enzyme assay kit was purchased from Roche (Roche Diagnostics GmbH, Germany) and Randox (Randox Laboratories Ltd., Crumlin Co. Antrim, UK). Brine shrimp eggs were purchased from an ornamental fish store, Colombo, Sri Lanka Sea water was collected from Galle Face Green, Colombo, Sri Lanka to conduct brine shrimp lethality assay. HPLC analysis was carried out with Shimadzu LC 10AS solvent delivery system equipped with UV/VIS detector Shimadzu SPD 10A and an integrator Shimadzu C-R8A (Shimadzu Corporation, Japan). LiChrosorb RP-18 (5 μm) column (2.1 x 150 mm) was used to obtain HPLC fingerprints. HPLC grade acetonitrile was used to prepare the solvent system. Centrifugation was carried out using Kubota 6500 (Kubota Corporation, Tokyo, Japan) and Biofuge D-37520 (Heraeus instruments) centrifuge. Cells were incubated at 37°C in humidified carbon dioxide incubator (SHEL LAB/ Sheldon Manufacturing Inc. Cornelius, OR 97113, USA) and ESCO (EQU/04-EHC) laminar flow (ESCO Micro Pte. Ltd, Singapore 486777) was used to carry out cell culture experiments. Cells were observed using Olympus (1X70-S1F2) inverted fluorescence microscope (Olympus Optical Co. Ltd. Japan). The photographs were taken using Scope photo microscope digital camera (MDC 200, USB 2.02M pixels, CCD chip). Deionized water was used for all experiments obtained from LABCONCO UV ultra-filtered water system (LABCONCO Corporation, Kansas city, Missouri 64132-2696).

### Plant Materials

Leaves of *S. parvifolia* (Heen Badulla) were collected from Bandaranayake Memorial Ayurvedic Research Institute premises, Navinna, Colombo, Sri Lanka. The plant was authenticated by the principal scientist Dr. Sudeepa Sugathadasa, at the Department of Botany, Bandaranayake Memorial Ayurvedic Research Institute, Navinna, Colombo, Sri Lanka. The voucher specimen was deposited at the same premises.

### Preparation of the Plant Extract

The air-dried leaves of *S. parvifolia* (250g) were powdered and extracted with deionized water (1 L). The contents were refluxed for 3 hours and filtered through a Whatmann filter paper (No 01). The resulting solution was freeze dried and stored at -20 ^o^C until used. Three individual extracts were prepared separately and lyophilized (*n* = 3). Each extract was characterized by total phenolic content using Folin- Ciocalteau method in triplicate [13].

### Instrumentation and Chromatographic Conditions for HPLC Fingerprints

Chromatographic separation was carried out at room temperature. Different chromatographic conditions (composition of the running solvents, detection wave lengths, and flow rates) were employed to optimize the separation and detection of peaks. The mobile phase consisted of 5% acetonitrile in 0.5% acetic acid at a flowrate of 1.5 mL/min was finally used to elute the substances present in the extract and detected at 235 nm after injection (100 μL) of the plant extract (1000 μg/mL).

### Cell Line

Human laryngeal carcinoma cell line, (HEp*-2*) was obtained from Medical Research Institute, Colombo 08, Sri Lanka.

### Preparation of Cells for Cytotoxic Experiments

MEM growth medium, composed of 10% fetal bovine serum (FBS), L-glutamine (3%), penicillin/streptomycin and sodium bicarbonate (7.7%) was employed to culture HEp-2 cells. The cells were seeded in 24-well plates at a density of 2 x 10^5^ cells per well for overnight in a humidified CO_2_ incubator. Lyophilized powder of *S. parvifolia* was dissolved in culture medium and freshly prepared extract was filtered through syringe filter (0.45 μm) for all experiments. Confluent monolayer was treated with different concentrations of *S. parvifolia* leaves for specified time period as described under each experiment. The cells were harvested and subjected to cytotoxicity experiments. A positive control with camptothecin (5 mM; 25 μL) and a negative control without the plant extract were used for all the experiments. All experiments were carried out at least in triplicate.

### MTT Assay

The MTT colorimetric assay was carried out as described by Mosmann [14]. Cells were seeded in 24 well plates as described earlier. The adhered cells were treated with *S. parvifolia* leave extracts at different concentrations for 12, 24 and 72 hours in humidified CO_2_ incubator at 37°C. Spent medium was replaced by fresh medium (2 mL) and MTT (5 mg/mL; 200 μL). The cells were incubated at 37°C for 3 hours and the medium was aspirated carefully. The formazan crystals were solubilized with 1.5 mL of 0.05 M HCl (in 2-propanol) and the absorbance was measured at 570 nm. The percentage cell viability was determined using the equation below.

$$ \%\mathrm{Cell}\ \mathrm{viability}=\left[\mathrm{Absorbance}\ \mathrm{of}\ \mathrm{the}\ \mathrm{sample}\right]/\left[\mathrm{Absorbance}\ \mathrm{of}\ \mathrm{the}\ \mathrm{untreated}\ \mathrm{cells}\right]\ \mathrm{X}\ 100 $$

EC_50_ was determined by linear regression analysis using the corresponding dose response curve against concentration.

### Lactate Dehydrogenase (LDH) Activity

Lactate dehydrogenase is released into the culture medium following loss of membrane integrity as a results in cell death. LDH assay was carried out to study the antiproliferative activity induced by *S. parvifolia*. Cells were seeded in 24-well plates and incubated overnight as described previously. Confluent monolayer was treated for 24 hours at different concentrations of the plant extract. Spent medium was collected and centrifuged. Cell lysate was prepared by sonicating the cells for 20 second after treatment with Trition-X-100 (0.1%; 1 mL). LDH activity of the spent medium and the lysate of all samples were determined using commercially available LDH assay kit according to the manufacturer’s instructions.

The percentage leakage was calculated as described below.

$$ \%\mathrm{Leakage}=\left[\mathrm{LDH}\ \mathrm{activity}\ \mathrm{in}\ \mathrm{the}\ \mathrm{supernatant}\right]/\left[\mathrm{Total}\ \mathrm{LDH}\ \mathrm{activity}\right]\ \mathrm{X}\ 100 $$

$$ \mathrm{Total}\ \mathrm{LDH}\ \mathrm{activity}=\mathrm{LDH}\ \mathrm{activity}\ \mathrm{of}\ \mathrm{the}\ \mathrm{supernatant}\ \mathrm{and}\ \mathrm{the}\ \mathrm{lysate} $$

### Sulforhodamine B (SRB) Assay

The SRB assay was performed as reported previously with slight modifications [15]. The principle of the assay is based on the ability of sulforhodamine B dye to bind electrostatically with basic amino acid residues of proteins. The protein content is proportional to the number of live cells adhered to the well. Cells were seeded in 24-well plates. Confluent monolayer was treated with different concentrations of the plant extract. After 24 hour of exposure, the cells were fixed with trichloroacetic acid (10%; 500 μL) and incubated at 4°C for one hour. The cells were dried completely after five washing cycles with deionized water. SRB (0.4% SRB dissolved in 1% CH3COOH; 500 μL) was then added to each well and allowed to stain for 30 minutes. The wells were subjected to five washing cycles again to remove unbound dye using 1% acetic acid (v/v) and air dried. The bound dye was dissolved with Tris base (10 mM; 500 μL) for 30 minutes using a shaker. The absorbance was measured at 564 nm using Tris base as the blank. The percentage viability was calculated according to the equation below.

$$ \%\mathrm{Cell}\ \mathrm{viability}=\left[\mathrm{Absorbance}\ \mathrm{of}\ \mathrm{treated}\ \mathrm{cells}\right]/\left[\mathrm{Absorbance}\ \mathrm{of}\ \mathrm{untreated}\ \mathrm{cells}\right]\ \mathrm{X}\ 100\% $$

### Measurement of Nitrite Levels

The supernatants obtained from MTT assay was used to determine nitrite levels. Griess reagent (1% sulfanilamide and 0.1 % N-1 naphthyl ethylenediamine dihydrochloride in 0.1 M hydrochloric acid; 400 μL) was mixed with the supernatant (400 μL) obtained after 24 hour treatment of the plant extract. The mixture was incubated for 10 minutes at room temperature and the absorbance was measured at 540 nm [16]. The standard curve was constructed using sodium nitrite (0.25 – 4.0 μg/mL).

### Brine Shrimp (*Artemia Salina*) Lethality Assay

Lethality assay for brine shrimps was performed as described previously to evaluate toxicity [17]. Ten nauplii were placed in petri dishes at different concentrations (25 – 4000 μg/mL) of *S. parvifolia extract* and made up to a final volume of 20 mL. The plates were maintained at room temperature for 24 hours under aeration. The surviving larvae were then counted. Experiment was carried out along with a negative control. The percentage lethality was calculated from the mean survival larvae treated with the plant extract compared to the control. The LC_50_ was calculated at a concentration of 50% deaths of *A. salina*.

$$ \%\mathrm{Lethality}=\left[\mathrm{Survival}\ \mathrm{larvae}\right]/\left[\mathrm{Survival}\ \mathrm{larvae}\ \mathrm{in}\ \mathrm{the}\ \mathrm{control}\right]\ \mathrm{X}\ 100 $$

### Morphological Observations

Cells were seeded in 24-well plates (2 x 10^5^ cells per well) and cultured overnight as described earlier. Adhered monolayer was treated with different concentrations (150, 300, 600 and 900 μg/mL) of the water extract of *S. parvifolia* leaves for 24 hours. The morphological changes of cells were detected by light microscopy.

Typical morphological features of apoptosis were further observed after Giemsa staining [18]. The cells (2 x 10^5^ per well) were treated at different concentrations (150, 300, 600 and 900 μg/mL) of *S. parvifolia* leave extract for 24 hours. Fresh medium (750 μL) was added to trypsinized cells and centrifuged at 3,000 rpm for 5 minutes. Cells were re-suspended in 50 μl cold PBS. The cell suspension (10 μL) was added to a glass slide and fixed with ethanol. A volume of diluted Geimsa (1:9; pH 7.2; 5 μL) was added to cells and left for 10 minutes. The slide was washed with deionized water and observed under light microscope using 200X magnification.

### Ethidium Bromide/Acridine Orange staining (EB/AO staining)

Mode of cell death and apoptotic features of nuclei were observed as described elsewhere [19]. The cells (2 x 10^5^) cultured in 24-well plates were treated at different concentrations (50, 100, 350 and 750 μg/mL) of *S. parvifolia* leaf extract for 24 hours. The supernatant was transferred to micro centrifuge tubes (2 mL). The adherent cells were detached with 1 mL of Trypsin-EDTA after incubating at 37°C for 2 minutes. The supernatant and the detached cells from the same sample were pooled together. The cell pellets obtained by centrifugation (2,000 rpm for 2 mins) were resuspended in 25 μL of cold PBS and 2 μL of EB/AO dye mix (100 μg/mL acridine orange and 100μg/mL ethidium bromide in PBS). Stained cell suspension (10 μL) was placed on a clean microscope slide and observed using Olympus (1X70-S1F2) inverted fluorescence microscope at 400X magnification. The images were captured using Scope photo microscope digital camera.

### Alkaline Comet Assay

Alkaline Comet assay (pH 13) was performed to detect DNA damage caused by the plant extract according to the protocol developed by Tice [20] with modifications. Microscope slides were prepared with normal melting agarose (1.0% in PBS). The cells cultured in 24-well plates (2 x 10^5^) were treated at different concentrations (150, 300, 600, 900 and 1000 μg/mL) of *S. parvifolia* leaves for 24 hours. The spent medium was transferred to separate micro centrifuge tubes. The adherent cells were detached with Trypsin-EDTA (1 mL). The supernatant and the detached cells were pooled together and centrifuged at 2,000 rpm for 3 minutes. Cells were collected and mixed with 100 μL of low melting point agarose (0.5 % in PBS at 37°C). The mixture (50 μL) was placed on a precoated slide with normal agarose (1% in PBS at 37°C) and immediately covered with a coverslip and left to solidify using ice packs. The cells were immersed in lysis buffer (2.5 M NaCl, 100 mM, EDTA, 10 mM Trizma base, 1% TritonX–100, 1% Sodium lauroyl sarcosine, 10% DMSO, pH 10.0; 500 mL) for 1.5 hours at 4 ^0^C. Then the slides were placed in ice cold alkaline buffer (5 M NaOH and 250 mM EDTA, pH>13; 500 mL) at 4°C for 30 minutes and subjected to electrophoresis for 30 minutes (0.7 V/cm, 300 mA) with freshly prepared pre-chilled electrophoresis buffer (5 M NaOH and 250 mM EDTA, pH>13; 750 mL). The slides were rinsed with pre-chilled neutralizing solution (0.4 M Triz, pH 7.5; 400 mL) and air dried. Absolute methanol was then added. The gels were stained with ethidium bromide (20 μg/mL; 30 μL) and observed using an inverted fluorescence microscope (400X). The photographs were taken using Scope Photo microscope digital camera. The comet length to head width ratio (Length of tail/width of head) was taken as the parameter to analyze comets. Fifty comets from each concentration were evaluated manually along with the positive and negative controls [21].

### Calculations and Statistics

All assays were carried out at least with three independent experiments and the results were presented as mean ± standard deviation (mean ± SD). Linear or nonlinear regression curves were used to calculate EC_50_ values for HEp-2 cells. The linear segment of the sigmoid curve of dose response curves was used to determine EC_50_ values. The statistical significance of differences between means was calculated applying Student’s t-test using Microsoft Excel. The value of p<0.05 was considered as statistically significant.

## Results

### Phenolic Content and HPLC Fingerprints

**Fig. 1:**
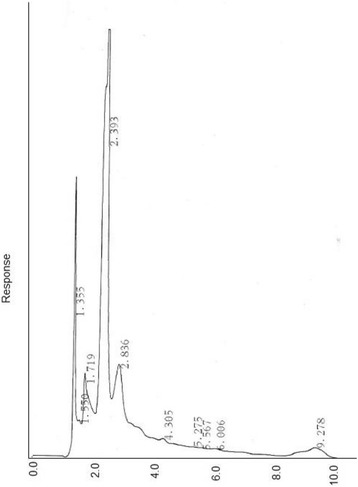
The HPLC fingerprints obtained at a concentration of 500 μg/mL. The Figure represents of a chromatogram from triplicate of individually prepared lyophilized plant extracts

The phenolic content of the *S. parvifolia* extract was 2.6±0.2% (W/W of the gallic acid equivalent) of the lyophilized sample. The HPLC fingerprints of *S. parvifolia* (500 μg/mL) showed four distinct peaks at retention times of 1.3, 1.7, 2.4 and 2.8 minutes with a broad peak at 9.3 minutes (Fig. 1).

### MTT Assay

**Fig. 2:**
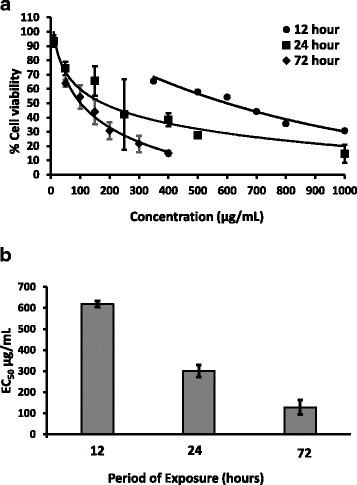
Mean of percentage cell viability (± SD) of HEp-2 cells determined by MTT assay (**a**) and EC_50_ values (± SD) after 12, 24 and 72 hour (**b**) exposure of *S parvifolia* Thw. leaves (*n* = 3)

Metabolically active cells reduce MTT (3-(4, 5-dimethythiazol-2-yl)-2, 5-diphenyl tetrazolium bromide) to an insoluble purple formazan crystals by mitochondrial dehydrogenase enzyme. Number of live cells is therefore proportional to the concentration of reduced MTT [13]. The EC_50_ values calculated using the respective dose response curves constructed between the percentage cell viability and the concentration of plant extract are depicted in Fig. 2a. The results demonstrated that, the cell viability decreased reciprocally with the concentration (Fig. 2a) and along the period of exposure (Fig. 2b).

### LDH Assay

**Fig. 3:**
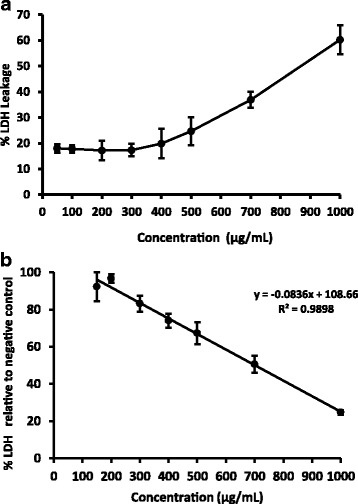
**a** Percentage LDH leakage (Mean ± SD; *n* = 3) of HEp-2 cells after 24 hour exposure of *Semecarpus parvifolia* Thw. at different concentrations; **b** The percentage LDH activity of the cell lysate of the treated cells to that of the negative control

The cell membrane integrity was reduced in cells treated with *S parvifolia* leaves in a dose dependent manner (Fig. 3). The EC_50_ value for percentage release of LDH after exposure to plant extract was calculated using two methods. The method 1 involved the percentage leakage of LDH to the total LDH activity (Fig. 3a) and the second method was the LDH activity remained in the cell lysate of cells treated with the plant extract to that of negative control (Fig. 3b). The percentage leakage of LDH of untreated cells and camptothecin (5 mM; 25 μL) over 24 hour was 14.5 ± 1.4% and 43.2 ± 1.9% respectively. The percentage LDH release was just above the level of negative control until the concentration reached up to 400 μg/mL (Fig. 3a and b) and a sharp increase was observed after 500 μg/mL (Fig. 3a).

### SRB Assay

**Fig. 4:**
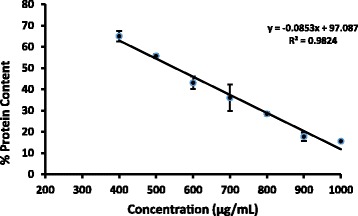
Percentage protein content (Mean ± SD; *n* = 3) of cell lysate to that of the untreated cells after 24 hour exposure of *Semecarpus parvifolia* Thw. leaves

The percentage protein content of the cell lysate of treated cells to that of negative control was decreased linearly along with the concentration (Fig. 4). The EC_50_ obtained for SRB assay is shown in the Table 1.

**Table 1:** The EC_50_ values for antiproliferative assays after 24 hour exposure of *Semecarpus parvifolia Thw*

Antiproliferative/Cytotoxicity assays	EC_50_ (μg/mL)
MTT	301.09 ± 28.2
LDH ^a^	864.9 ± 36.6
LDH ^b^	702.2 ± 16.0
SRB	554.8 ± 14.1
Brine Shrimp	>4000

^a^Calculated by Percentage leakage of LDH in the medium to that of total

^b^Calculated by LDH activity of cell lysate to that of negative control

### Nitric Oxide Levels

**Fig. 5:**
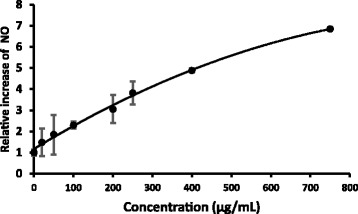
Increase in nitric oxide levels at different concentrations of *Semecarpus parvifolia* leaves against HEp– 2 cells (mean ± SD; *n* = 3). Nitric oxide content of the treated cells relative to that of untreated cells was used to calculate the relative increase of NO

Nitric oxide levels in the spent medium were increased linearly with the concentration (Fig. 5) after 24 hour treatment with *S. parvifolia* .

### Brine Shrimp Lethality Assay

No toxicity against brine shrimps was observed even at a concentration of 4000 μg/mL of *S. parvifolia* leaf extract.

### Morphological Analysis

**Fig. 6:**
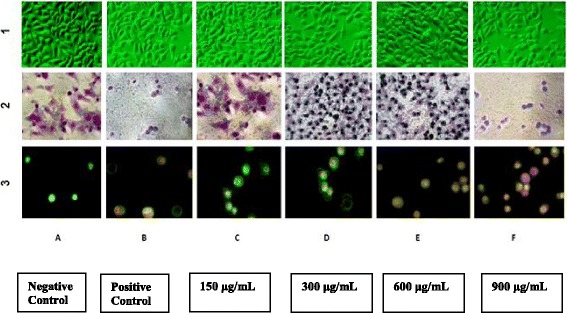
Row 1: Representative light micrographs of HEp-2 cells (Magnification 100X). Row 2: after staining with Giemsa of HEp-2cells (Magnification 200X). Row 3: Represents Comet formation by Single cell gel electrophoresis of HEp-2 cells (Magnification 400X). **a** Negative control with untreated cells; **b** Positive control with Camptothecin (5mM; 25 μL); **c**, **d**, **e** and **f** are HEp-2 cells treated with the water extract of *Semecarpus parvifolia* leaves at 150, 300, 600 and 900 μg/mL concentrations for 24 hours respectively

The morphological changes of HEp– 2 cells were observed by light microscopy and fluorescent microscopy following 24 hour post treatment with the plant extract (Fig. 6). Spindle shaped structures had been transformed to irregular shapes with condensed nuclei contrast to untreated cells (Fig. 6-1 and 2). The adherent cells become rounded, shirked and detached from the well plate in a concentration dependent manner. Cell free areas were visible in the wells at higher concentrations as observed for the positive control (Fig. 6-1b). Giemsa stained cells demonstrated apoptotic features at concentrations higher than 150 μg/mL and morphology were similar to the cells treated with positive control (Fig. 6-2b). EB/AO stained cells were identified as described by Ribble et al [19]. Untreated cells showed normal nuclei, stained with green florescence (Fig. 6-3-a). Fragmented cells with green and green orange nuclei of early apoptotic and red orange nuclei with necrotic cells were observed after EB/AO staining. Accordingly, positive control and cells treated with the plant extracts showed apoptotic cells (Fig. 6-3, b, c, d, e, and f). Majority of necrotic cells with red orange nuclei were visible at 900 μg/mL (Fig. 6-3-f). Living cells, stained with green florescence were observed with untreated cells (Fig. 6-3 a) and early apoptotic cells were stained as fragmented green nucleus with green spots or observed as green crescents (Fig. 6-3- d). The late apoptotic cells were visible as green yellow round shape with orange-yellow color chromatins inside and red color showed the necrotic cells. Concentration dependent increase in induction of apoptosis was observed after treatment with the plant extract.

### Comet Assay

**Fig. 7:**
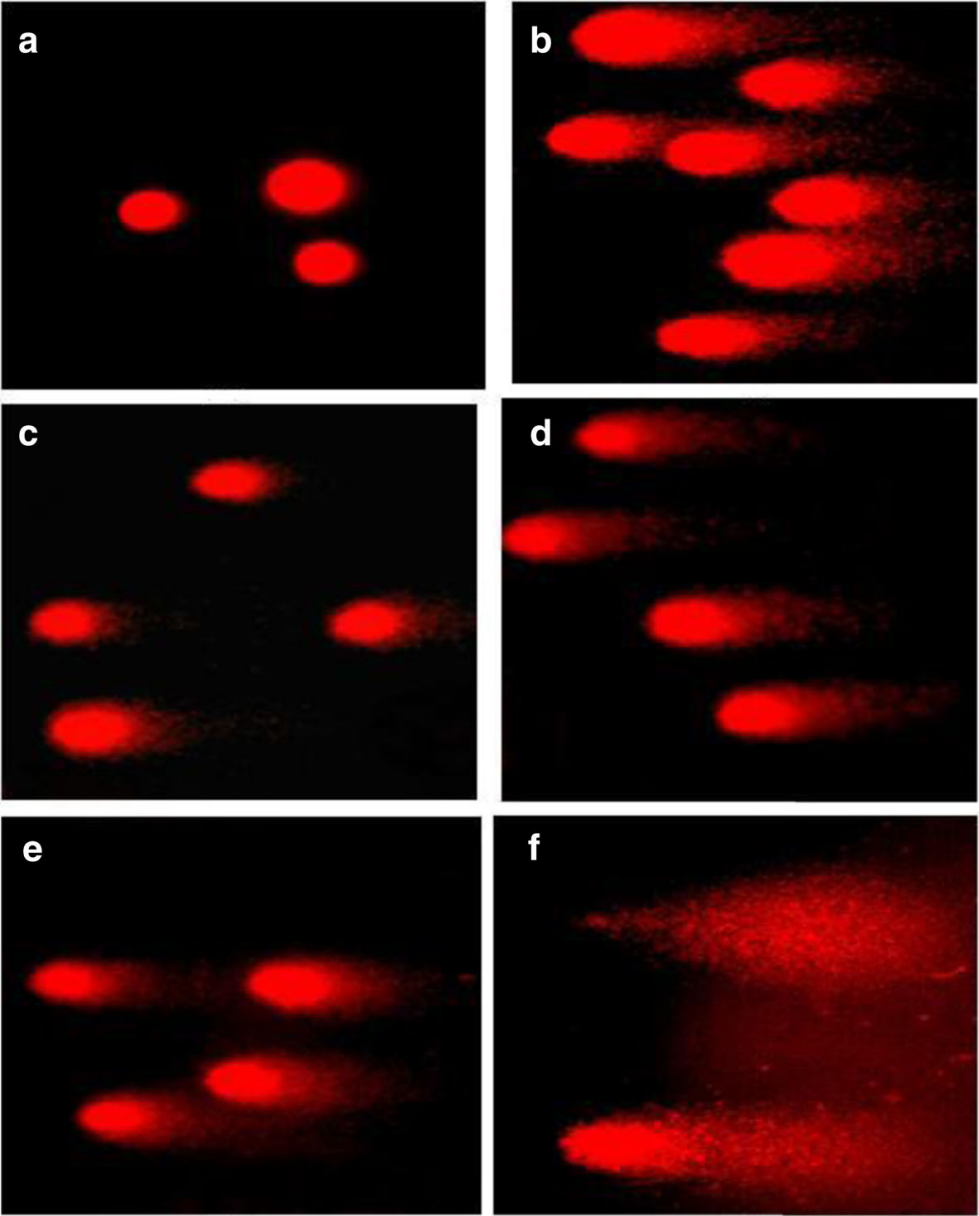
Comet formation by single cell gel electrophoresis of HEp*-*2 cells after treatment at different concentrations of the water extract of *Semecarpus parvifolia* leaves; (**a**) untreated, (**b**) Cyclohexamide as the positive control (5 mM; 25 μL), 150 (**c**), 300 (**d**), 600 (**e**), 900 μg/mL (**f**) of the plant extract (400X)

**Fig. 8:**
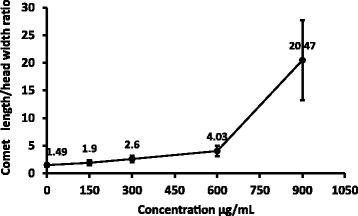
The ratio of comet length to head width (M ± SD; *n* = 50) of HEp-2 cells treated with *Semecarpus parvifolia* Thw.at different concentrations

Comet assay (single-cell gel electrophoresis) was used to detect primary DNA damage [22]. Figure 7 shows the migration of fragmented DNA at different concentrations of the plant extract. A significant increase (P<0.01) in tail length to head width ratio was demonstrated at 900 μg/mL compared to all the concentrations investigated including negative control [Fig. 8]. Camptothecin which was used as the positive control (5 mM; 25 μL) showed a value of 3.1 ± 0.9 for comet length to head ratio.

## Discussion

Poly herbal drugs are used in Ayurveda and Traditional systems of medicine. *S. parvifolia* is one of the components in poly herbal drugs used in the treatment of cancer by traditional doctors. Aqueous extract of *S. parvifolia* leaves has been reported to possess scavenging activity against reactive oxygen and nitrogen species including hydroxyl, superoxide and nitric oxide [12]. Some plants which have high antioxidant activity are capable to inhibit cell proliferation [23]. Based on its capacity to scavenge free radicals, we investigated the ability to inhibit cell proliferation and induction of apoptosis by the water extract of *S. parvifolia* leaves.

The most extensively studied plant in *Semecarpus* genus on antproliferative activity is *S anacardium* [24]. Semecarpus Lehyam, a paste composed of *S anacardium* nut is used in Siddha medicine to treat breast cancer [8]. The water extract of Semecarpus Lehyam has retained almost 100% cell viability at a concentration of 60 μg/mL while the present study demonstrated that there was only 74±4.7% cell viability for the water extract of *S*. *parvifolia* leaves at a concentration of 50 μg/mL. It has been reported that, oil of *S anacardium* nut and hydroalcoholic extract have cytotoxic activity against different cell lines [6, 25]. Further *S anacardium* has an antitumor efficacy in rats induced with aflatoxin B1 mediated hepatocellular carcinoma [26].

Present study demonstrated a concentration dependent increase in NO levels in the culture medium after treatment with *S*. *parvifolia.* Accumulation of NO activates kinases by phosphorylation of p53 converting it to an active form [27]. Activated P53 induces caspases leading to chromatin condensation and DNA fragmentation [28]. Previous studies report, the involvement of plants in NO mediated apoptosis in HepG-2 and A375 cells [16, 29]. The levels of nitric oxide produced were significantly high at concentrations higher than 100 μg/mL of *S*. *parvifolia*. The increase of NO levels in the medium was several folds (1.5-7.0) compared to the negative control for all concentrations investigated. The present study suggests that the excessive production of NO synthesis stimulated by *S*. *parvifolia* leave extract may direct the upregulation of apoptotic cell death. The loss of plasma membrane integrity and mitochondrial function as a result of initiation of cell death were shown by *S. parvifolia* in a concentration and time dependent manner. Decrease in protein content observed by SRB assay further confirmed the induction of cell death with the plant extract. Morphological observation found with light microscopy showed shrinkage of cells and signs of apoptosis. Cells stained with Giemsa, further exhibited characteristic changes in cell morphology indicating the apoptotic cell death in HEp*-*2 cells after treating the cells with the plant extract. Staining with EB/AO dye mix determines the membrane integrity of a cell, based on the uptake or exclusion of a dye from the cell [30]. The distinct formation of crescent or ring like structures induced by *S. parvifolia* proved that the condensation started peripherally along the nuclear membrane [31]. Appearance of apoptotic cells increased with the concentration of the plant extract which was identical to that of camptothecin treated cells.

DNA damage caused by apoptotic cell death was ascertained by comet assay. A typical measurement to detect the DNA damage is the ratio between the comet length and head width [20]. Untreated cells showed an oval shape but minimum or no migration of fragmented DNA. Comet assay confirmed that less toxicity on HEp–2 cells at lower concentrations. However a dose dependent increase was observed in DNA strand breaks. The ratio of the comet length to head width increased linearly up to 600 μg/mL with a sharp increase at 900 μg/mL. The level of fragmented DNA was consistent with the LDH release. As described by Dias et al, the comets of fragmented DNA induced by *S. parvifolia* could be related to the induction of apoptosis [32].

The brine shrimp cytotoxicity assay is considered as a convenient probe for preliminary assessment of toxicity and antitumor activity detection of plants, heavy metals and pesticides [17, 33]. The brine shrimps were able to survive even at the highest concentration (4 mg/mL) of *S. parvifolia* employed. Thus it reflects that the plant does not show a toxicity on normal multicellular system.

## Conclusions

The water extract of *Semecarpus parvifolia* Thw leaves attenuates cell proliferation of HEp-2 cells. Typical structural changes and DNA damage demonstrate that the cell death occurs via apoptosis and NO plays an important role in the mechanism of action. Since this plant extract is used in the preparation of poly herbal formulations in cancer therapy in Traditional medicine, the efficacy remains to be confirmed further by in vivo models and clinical studies.

## Abbreviations

EB/AO: Ethidium bromide/acridine orange
HPLC: High performance liquid chromatography
LDH: Lactate dehydrogenase
MTT: 3-(4, 5-dimethythiazol-2-yl)-2, 5-diphenyl tetrazolium bromide
SRB: Sulforhodamine B

## References

[1] American Cancer Society. Cancer Facts & Figures 2016, Atlanta. GA: American Cancer Society. p. 2016.

[2] Sharma S, Kelly TK, Jones PA (2010). Epigenetics in cancer. Carcinogenesis..

[3] Pan L, Chai H, Kinghorn A (2010). The continuing search for antitumor agents from higher plants. Phytochem Lett..

[4] Cragg G, Grothaus P, Newman D (2009). Impact of natural products on developing new anti-cancer agents. Chem Rev..

[5] Cragg GM, Newman DJ (2013). Natural products: A continuing source of novel drug leads. Biochim Biophys Acta..

[6] Premalatha B (2000). *Semecarpus anacardium Linn*. nuts-a boon in alternative medicine. Indian J Exp Biol..

[7] Semalty M, Semalty A, Badola A, Joshi GP, Rawat MSM (2010). *Semecarpus Anacardium Linn.*:A review. Pharmacogn Rev..

[8] Sowmyalakshmi S, Nur-e-Alam M, Akbarsha M, Thirugnanam S, Rohr J, Chendil D (2005). Investigation on Semecarpus Lehyam a Siddha medicine for breast cancer. Planta..

[9] Jayaweera DMA, Senaratna L (2006). Medicinal plants (indigenous and exotic) used in Ceylon.

[10] Sujatha V, Sachdanandam P (2002). Recuperative effect of *Semecarpus anacardium* linn. nut milk extract on carbohydrate metabolizing enzymes in experimental mammary carcinoma-bearing rats. Phytother Res..

[11] Joseph JP, Raval SK, Sadariya KA, Jhala M, Kumar P (2013). Anti-cancerous efficacy of ayurvedic milk extract of *Semecarpus anacardium* nuts on hepatocellular carcinoma in Wistar rats. Afr J of Tradit Complement Altern Med..

[12] Jayarathna DDPP and Soysa SSSBDP. Total phenolic content and antioxidant activity of the water extract of Semecarpus parvifolia (Heen Badulla). Proceedings of the 68th Annual Sessions of Sri Lanka Association for Advancement of Science. 66**,** 2012**.**

[13] Silva IK, Soysa P (2011). Evaluation of phytochemical composition and antioxidant capacity of a decoction containing *Adenanthera pavonina L*. and *Thespesia populnea L*. Pharmacogn Mag..

[14] Mosmann T (1983). Rapid colorimetric assay for cellular growth and survival: Application to proliferation and cytotoxicity assays. J of Immunol Methods..

[15] Voigt W, Sulforhodamine B (2005). assay and chemosensitivity. Methods Mol Med..

[16] Wageesha NDA, Soysa P, Atthanayake K, Choudhary MI, Ekanayake M. A traditional poly herbal medicine “Le Pana Guliya” induces apoptosis in HepG2 and HeLa cells but not in CC1 cells: an in vitro assessment. Chem Cent J. 2017; 11(1), DOI: 10.1186/s13065-016-0234-4CCJO-D-16-00207.1 2017. 10.1186/s13065-016-0234-4 PMC5215177 28101129

[17] Meyer BN, Ferrigni NR, Putnam JE, Jacobsen LB, Nichols DE, McLaughlin J (1982). Brine Shrimp: A convenient general bioassay for active plant constituents. Planta Med..

[18] Yu Z, Zhang T, Zhou F, Xiao X, Ding X, He H, Rang J, Quan M, Wang T, Zuo M, Xia L (2015). Anticancer Activity of Saponins from Allium chinense against the B16 Melanoma and 4T1 Breast Carcinoma Cell. Evid Based Complement Alternat Med..

[19] Ribble D, Goldstein NB, Norris DA, Shellman YGA (2005). simple technique for quantifying apoptosis in 96-well plates. BMC Biotechnology..

[20] Tice RR, Agurell E, Anderson D, Burlinson B, Hartmann A, Kobayashi H, Miyamae Y, Rojas E, Ryu J-C, Sasaki YF (2000). Single cell gel/comet assay: Guidelines for in vitro and in vivo genetic toxicology testing. Environ Mol Mutagen..

[21] McCarthy PJ, Sweetman SF, McKenna PG, McKelvey-Martin VJ (1997). Evaluation of manual and image analysis quantification of DNA damage in the alkaline comet assay. Mutagenesis..

[22] Angeli J, Barcelos GR, Serpeloni JM, Barbosa Jr F, Nersesyan A, Mantovani MS (2009). Evaluation of the genotoxic and anti-genotoxic activities of silybin in human hepatoma cells (HepG2). Mutagenesis..

[23] Jiménez-Estrada M, Velázquez-Contreras C, Garibay-Escobar A, Sierras-Canchola D, Lapizco-Vázquez R, Ortiz-Sandoval C, Burgos-Hernández A, Robles-Zepeda RE: *In vitro* antioxidant and antiproliferative activities of plants of the ethnopharmacopeia from northwest of Mexico. BMC Complem Altern Med. 2013, 13: 12. 10.1186/1472-6882-13-12. 10.1186/1472-6882-13-12 PMC3547710 23305162

[24] Mathivadhani P, Shanthi P, Sachdanandam P (2007). Apoptotic effect of *Semecarpus anacardium* nut extract on T47D breast cancer cell line. Cell Biology International..

[25] Chakraborty S, Roy M, Taraphdar AK, Bhattacharya RK (2004). Cytotoxic effect of root extract of Tiliacora racemosa and oil of *Semecarpus anacardium* nut in human tumour cells. Phytotherapy Research..

[26] Premalatha B, Muthulakshmi V, Sachdanandam P (1999). Anticancer potency of the milk extract of *Semecarpus anacardium* Linn. nuts against aflatoxin B1 mediated hepatocellular carcinoma bearing wistar rats with reference to tumour marker enzymes. Phytother Res..

[27] Brüne B (2003). Nitric oxide: NO apoptosis or turning it ON?. Cell Death Differ..

[28] Brüne B, von Knethen A, Sandau KB (1998). Nitric oxide and its role in apoptosis. Eur J Pharmacol..

[29] Kim MY (2012). Nitric oxide triggers apoptosis in A375 human melanoma cells treated with capsaicin and resveratrol. Mol Med Rep..

[30] Ariffin SHZ, Omar WHW, Ariffin ZZ, Safian MF, Senafi S, Wahab RMA (2009). Intrinsic anticarcinogenic effects of *Piper sarmentosum* ethanolic extract on a human hepatoma cell line. Cancer Cell Int..

[31] Ziegler U, Groscurth P (2004). Morphological features of cell death. News Physiol Sci..

[32] Dias E, Louro H, Pinto M, Antunes S, Pereira P, Silva MJ. Genotoxicity of Microcystin-LR in in vitro and in vivo experimental models. Biomed Res Int. 2014; 10.1155/2014/949521, 2014. 10.1155/2014/949521 PMC4052155 24955368

[33] Huang JM, Nakade K, Kondo M, Yang CS, Fukuyama Y (2002). Brine shrimp lethality test active constituents and new highly oxygenated seco-prezizaane-type sesquiterpenes from *Illicium merrillianum*. Chem Pharm Bull (Tokyo)..

